# Glyphosate Bioremediation Facilitated by *Serratia ureilytica*-Derived Biosurfactants Using Amazonian Biodiversity: Genomic Insights and Adsorption Dynamics

**DOI:** 10.3390/jox16020062

**Published:** 2026-04-04

**Authors:** Kleyson Willames da Silva, Emilly Cruz da Silva, Giulian César da Silva Sá, Joane de Almeida Alves, Darlisson de Alexandria Santos, Alexandre Orsato, Karoline Leite, Dante Santos da Silva, Adriano Richard Santos da Silva, Zanderluce Gomes Luis, Flavia Karoliny Araujo dos Santos, José Augusto Pires Bitencourt, Cristina Maria Quintella, Pamela Dias Rodrigues, Doumit Camilios-Neto, Paul R. Race, James E. M. Stach, Sidnei Cerqueira dos Santos

**Affiliations:** 1 Laboratório de Bioensaios e Bioprocessos (L@βio), Programa de Pós-Graduação em Química, Universidade Federal do Sul e Sudeste do Pará (Unifesspa), Campus III, Maraba 68500-000, PA, Brazil; silvawillames1083@gmail.com (K.W.d.S.); joaneaa@unifesspa.edu.br (J.d.A.A.); 2 Laboratório de Estudos de Materiais para Adsorção e Sensoriamento (LEMAS), Núcleo de Pesquisas Energéticas (NUPENERG), Universidade Federal de Roraima (UFRR), Campus Paricarana, Boa Vista 69310-000, RR, Brazil; emilly.silva@ufrr.br (E.C.d.S.); danielysilva0209@gmail.com (D.S.d.S.); adrianorichard06@gmail.com (A.R.S.d.S.); 3 Laboratório de Síntese Orgânica e Aplicações Biotecnológicas, Departamento de Química Fundamental, Universidade Federal de Pernambuco (UFPE), Recife 50740-560, PE, Brazil; darlisson.santos@ufpe.br; 4 Laboratório de Síntese de Moléculas Medicinais, Departamento de Química, Universidade Estadual de Londrina (UEL), Londrina 86057-970, PR, Brazil; orsato@uel.br (A.O.); karolineleitte17@gmail.com (K.L.); 5 Laboratório de Botânica e Ecologia, Universidade Federal do Sul e Sudeste do Pará (Unifesspa), Campus III, Maraba 68500-000, PA, Brazil; zan.gomes@unifesspa.edu.br (Z.G.L.); flavia.karol@unifesspa.edu.br (F.K.A.d.S.); 6 Instituto Tecnológico Vale (ITV), Belem 66055-090, PA, Brazil; jose.augusto.bitencourt@itv.org; 7 Departamento de Química Inorgânica e Geral, Instituto de Química, Universidade Federal da Bahia (UFBA), Salvador 40170-115, BA, Brazil; cris5000tina@gmail.com (C.M.Q.); pamelarodrigues.ufba@gmail.com (P.D.R.); 8 Laboratório de Bioquímica e Biotecnologia de Microrganismos, Departamento de Bioquímica e Biotecnologia, Universidade Estadual de Londrina (UEL), Londrina 86057-970, PA, Brazil; camiliosneto@uel.br; 9 School of Natural and Environmental Sciences, Newcastle University, Newcastle upon Tyne NE1 7RU, UK; paul.race1@newcastle.ac.uk (P.R.R.); jem.stach@newcastle.ac.uk (J.E.M.S.)

**Keywords:** agro-industrial wastes, Amazonian resources, circular bioeconomy, glyphosate remediation, microbial biosurfactant, *Serratia ureilytica*

## Abstract

The pervasive environmental dispersal of glyphosate has established this herbicide as a dominant anthropogenic xenobiotic, necessitating advanced bioremediation strategies to restore soil integrity. This study assessed the bioremediation efficacy of biosurfactants produced by *Serratia ureilytica* BM01-BS in glyphosate-contaminated soils, establishing their adsorption dynamics and ecotoxicological safety. The strain *S. ureilytica* BM01-BS gave a biosurfactant yield of 3.7 g·L^−1^ with promising surface properties, utilizing babassu (*Attalea speciosa*) waste as the sole nutrient source. Whole-Genome Sequencing and Biosynthetic Gene Cluster mining identified a Nonribosomal Peptide Synthetase cluster homologous to rhizomide-type lipopeptides responsible for biosurfactant production. Bioremediation assays in glyphosate-contaminated soils demonstrated a removal efficiency exceeding 95% in approximately 60 min, outperforming the synthetic surfactant SDS (20–30% efficiency). Kinetic and isothermal modeling suggest that the bioremediation process is governed by chemisorption, adhering to a pseudo-second-order model (R^2^ = 0.998) with a maximum adsorption capacity of 845 µg·kg^−1^. Fourier-Transform Infrared spectroscopy confirmed that the biosurfactant effectively removes glyphosate and restores the soil’s mineral integrity, as evidenced by the complete disappearance of glyphosate-associated phosphonic and carboxylic bands. Ecotoxicological assessments verified the environmental safety of the bioremediation process. These findings position the BM01-BS biosurfactant as a sustainable, biodiversity-based adjuvant for enhancing ecological resilience in glyphosate-impacted landscapes.

## 1. Introduction

Glyphosate [N-(phosphonomethyl)glycine] is recognized as the most pervasive anthropogenic xenobiotic in modern agriculture, serving as a broad-spectrum post-emergent herbicide with an estimated global application exceeding 8.6 billion kilograms over the last four decades [1]. Despite its efficacy in weed management, escalating environmental concentrations of glyphosate in soil and aquatic systems represent a significant environmental threat [2].

The chemical persistence of glyphosate is fundamentally rooted in the profound thermodynamic stability of its carbon-phosphorus (C–P) bond, ensuring that the phosphonomethyl moiety remains intact under varying environmental stressors, contributing significantly to the herbicide’s status as a persistent anthropogenic xenobiotic [3]. However, the environmental behavior and longevity of glyphosate are also extensively regulated by its strong adsorption affinity for mineral surfaces and soil organic matter. This intimate interaction with the soil matrix often leads to the sequestration of the herbicide within mineral lattices, making it highly recalcitrant to both natural degradation pathways and standard remediation techniques [4]. Beyond environmental disruption, glyphosate legacy includes significant risks to human health, acting as an endocrine disruptor and inducer of oxidative stress [1,5]. Such molecular resilience necessitates the application of bio-rational, innovative amphiphilic xenometabolites capable of disrupting these soil-xenobiotic complexes to facilitate mobilization and subsequent bioremediation of the herbicide.

Simultaneously, the accumulation of agro-industrial wastes, notably those derived from the babassu palm (*Attalea speciosa* Mart.) processing in the Brazilian Amazon, has emerged as a parallel environmental challenge [6]. Regulatory frameworks, including Brazil’s Law 12,305/2010, have intensified the urgency for the valorization of such residual biomass within a circular bioeconomy. In this context, integrating waste valorization with xenobiotic bioremediation offers a strategic dual-benefit approach. Microbial biosurfactants—amphiphilic secondary metabolites capable of reducing surface and interfacial tensions and forming micellar complexes with organic pollutants—have emerged as promising agents for mobilizing and solubilizing persistent soil contaminants [7]. Unlike synthetic surfactants, these biological counterparts offer superior biocompatibility and biodegradability, positioning them as ideal candidates for “green” remediation of herbicide-contaminated sites [8].

Among biosurfactant-producing bacteria, the genus *Serratia* is recognized for its metabolic versatility in degrading complex xenobiotics, such as pesticides, pharmaceuticals, and industrial chemicals [9]. This study focuses on a strategically isolated strain, *Serratia ureilytica* BM01-BS, sourced from the extreme microbial consortia of a bauxite mine in Pará, Brazil. This environment, characterized by high mineral stress, serves as a natural selector for robust xenometabolic pathways. While microbial remediation has been previously explored [10,11], a significant knowledge gap persists regarding the use of Amazonian endemic waste as the sole nutritional platform for producing specialized biosurfactants targeted at glyphosate suppression.

In this study, we investigated the glyphosate-bioremediation efficacy of glycolipid biosurfactants produced by *S. ureilytica* BM01-BS, using babassu waste as a renewable feedstock, establishing the adsorption dynamics and assessing the ecotoxicological safety using lettuce (*Lactuca sativa* L.) as a biological indicator. High-resolution Whole-Genome Sequencing (WGS) was employed to elucidate the Biosynthetic Gene Clusters (BGCs) responsible for biosurfactant production, specifically targeting lipopeptide structures. By integrating genomic insights with applied environmental chemistry, this research offers a scalable framework for restoring ecological resilience in herbicide-impacted landscapes.

## 2. Materials and Methods

### 2.1. Ethics Approval

No unexpected or unusually severe safety hazards were encountered that would necessitate approval from an ethics committee.

### 2.2. Biological Resources

*Serratia ureilytica* BM01-BS was strategically isolated from the superficial soil layers of an active bauxite mining site in Marabá, Pará, Brazil. This environment was selected due to its heightened mineral stress conditions, which are expected to favor the development of robust xenometabolic pathways. The strain exhibits phenotypic characteristics typical of a Gram-negative bacillus and is preserved in a 20% glycerol solution at −20 °C at the Laboratory of Bio-test and Bio-processes (L@βio) from Campus III of Unifesspa. Legal compliance was ensured through registration with the Brazilian National Management System of Genetic Heritage (SisGen, registration n. A4DA401).

Agro-industrial waste from babassu palm (*Attalea speciosa* Mart.) (Arecales: Arecaceae) processing (predominantly fruit peels and exhausted pulp) was sourced from the Associação do Movimento Interestadual das Quebradeiras de Coco Babaçu in São Domingos do Araguaia, Pará, Brazil (SisGen n. AB70C5B). To achieve a standardized fermentation feedstock, the waste underwent a rigorous pre-treatment: it was extensively washed with running tap water to remove exogenous debris and subsequently dehydrated in a forced-ventilation oven (SP-102/64, SP Labor, Presidente Prudente, SP, Brazil) at 45 ± 2 °C until a constant weight was achieved. The dried lignocellulosic matrix was then ground using a Willey-type knife mill (SL-31, Solab, São Paulo, SP, Brazil) to achieve a particle size of approximately 0.07 mm. The carbohydrate content of babassu waste was determined following the methodology outlined by Feio et al. [12].

### 2.3. High-Resolution Genome Sequencing and Bioinformatic Annotation

*Serratia ureilytica* BM01-BS cells were lyophilized and sent for genomic DNA extraction, library preparation, and sequencing at the Vale Institute of Technology for Sustainable Development (ITV DS). Genomic DNA was extracted using the DNeasy PowerSoil Pro Kit (Qiagen^®^, Venlo, LI, The Netherlands), yielding a concentration of 210 ng·µL^−1^. The quality and concentration of the DNA were assessed using 1% agarose gel electrophoresis (Thermo Fisher Scientific™, Waltham, MA, USA) and quantified with a Qubit 3.0 fluorometer employing the 1X dsDNA BR Assay Kit (Thermo Fisher Scientific™).

DNA libraries were constructed using the Rapid Barcoding Kit (Oxford Nanopore Technologies; ONT, New York, NY, USA), and sequencing was performed on the PromethION 2 Solo (ONT) platform with R10.4.1 flow cells, utilizing two cells with a total of 1120 pores each. Initial sequencing output revealed low throughput, necessitating resequencing, which generated a cumulative total of 7 Gb of raw data. The bioinformatic processing included read trimming with Porechop (version 0.2.4) and quality filtering using Filtlong (version 0.2.1), with reads shorter than 3000 bp excluded.

Library statistics were analyzed with NanoStat (version 1.6.0) [13]. Subsequent assembly of reads was conducted by partitioning the ONT data into four maximally independent subsets, followed by assembly with Flye (version 1.9.1-b1784) [14], Raven (version 1.8.1) [15], NECAT (version 0.0.1) [16], and Miniasm (version 0.3.r179) combined with Minipolish (version 0.1.3), all integrated using the Autocycler (version 0.1.2) framework.

Taxonomic validation was performed using the Genome Taxonomy Database (GTDB-Tk version 2.2.5) [17], confirming a 97% Average Nucleotide Identity (ANI) with *S. ureilytica* CCUG:50595 (https://www.ncbi.nlm.nih.gov/nuccore/JABXOF000000000.1, accessed on 10 March 2026). The closest reference was GCF_013375155.1 (https://www.ncbi.nlm.nih.gov/datasets/genome/GCF_013375155.1/, accessed on 10 March 2026). The consensus assembly underwent further correction using Medaka (version 2.2.1). Annotation of the assemblies was conducted through the NCBI prokaryotic genome annotation pipeline (2025-05-06.build7983) [18]. The final consensus assembly was evaluated for completeness using BUSCO (version 5.2.2) [19]. Furthermore, Biosynthetic Gene Clusters (BGCs) were identified via AntiSmash 8.0 [20], targeting Nonribosomal Peptide Synthetase (NRPS) clusters. Genome images were generated with GenoVi (version 0.4.3) [21]. Protein homology searches were carried out using BLASTP (version 2.17.0) and HMMER (version 3.3.1) methodologies.

### 2.4. Sustainable Biosynthesis of Biosurfactant

Biosurfactant production was conducted in Erlenmeyer flasks containing 100 mL of sterilized mineral saline medium (MSM) formulated with the following components (g·L^−1^): K_2_HPO_4_ (4.0), Na_2_HPO_4_ (1.5), NaNO_3_ (1.0), MgSO_4_·7H_2_O (0.2), CaCl_2_·2H_2_O (0.02), and FeCl_3_·6H_2_O (0.02), as per the protocol established by Bodour et al. [22]. The pH was adjusted to 7.0 prior to sterilization. Babassu waste served as the sole carbon source at a 2% (*w*/*v*) concentration. The flasks were inoculated with a 5% standardized bacterial suspension (1.5 × 10^8^ cells·mL^−1^; optical density at 600 nm between 0.6 and 0.8, measured with a Biovera Bel V-M5 spectrophotometer, Wildberg, BW, Germany) and were incubated at 35 °C under continuous orbital shaking at 180 rpm for 120 h.

### 2.5. Purification of the Biosurfactant

Post-fermentation, the cell-free broth (CFB) was obtained through centrifugation (4000 rpm at 4 °C for 20 min; centrifuge SL-700, Solab, Aberdeen, AB, Scotland) and subsequently acidified to pH 2.0 using hydrochloric acid (6 N). Following overnight maturation period at 4 °C, the resulting precipitate was recovered via triplicate extraction using a chloroform/methanol (3:1, *v*/*v*) solvent system. This mixture was mechanically stirred and allowed to phase-separate over 24 h. The organic phase was then concentrated via rotary evaporation (rotary evaporator LGI-52CS-1, Scientific, São Paulo, SP, Brazil) and desiccated until a stable weight was achieved, resulting in the biosurfactant purified.

### 2.6. Biosurfactant Characterization

#### 2.6.1. Physicochemical Properties

The efficacy of the biosurfactant (10 mg·mL^−1^; suspended in distilled water) was assessed through the emulsification index (EI_24_) against mineral oil, with 1% sodium dodecyl sulfate (SDS; Dinamica Química Contemporânea LTDA, Indaiatuba, SP, Brazil) serving as the control [23]. Surface tension measurements were performed using a DataPhysics Oca15 plus tensiometer (Filderstadt, BW, Germany) via the drop shape analysis method, employing distilled water as the control. Interfacial tension was determined by the pendant drop method, using semi-synthetic lubricating oil (15W-40, Lubrax, Rio de Janeiro, RJ, Brazil) as the immiscible phase [24]. The Point of Zero Charge (PZC) was evaluated in a 0.01 M NaCl electrolyte across a pH gradient, with final pH measurements performed using a Hanna Instruments HI2210 pH meter (Woonsocket, RI, USA). The PZC was identified as the pH where the initial and final pH readings intersect, indicating surface charge neutrality [25].

#### 2.6.2. Structural Analysis

The isolated biosurfactant was characterized via Fourier-Transform Infrared Spectroscopy (FTIR) and Electrospray Ionization Mass Spectrometry (ESI-MS). FTIR spectra were recorded on a Bruker Vertex 70 FT-IR spectrometer (Bruker Daltonics, Billerica, MA, USA), covering a range from 4000 to 400 cm^−1^, and analyzed with OriginPro 8.0 software. ESI-MS analysis was performed using a QTOF mass spectrometer (Bruker Daltonics) equipped with direct injection. Mass spectra were obtained in both positive and negative ion modes over a full scan range from 50 to 1500 *m*/*z*. Instrument parameters included a capillary voltage of 3.8 kV, nitrogen as the nebulizer gas with a flow rate of 4.0 L·min^−1^, a gas temperature of 200 °C, and an ion energy of 5.0 eV.

### 2.7. Bioremediation Assays for Glyphosate-Contaminated Soils

Adsorption dynamics were evaluated using soil collected from Maraba, PA, Brazil, prepared by sieving through a 4.75 mm mesh. Soil samples (10 g each) were weighed, washed with distilled water, dried in an oven at 100 ± 2 °C to a constant weight, and subsequently added to a glyphosate solution to achieve specific xenobiotic loads (50, 100, 300, and 500 µg·kg^−1^; fresh weight based), followed by a seven-day equilibrium period. Control experiments utilized 1% SDS as a positive control, alongside uninoculated MSM as a negative control. The biosurfactant concentration of 500 µg·L^−1^ was selected based on Ordinance No. 2914/2011 from the Brazilian Ministry of Health regarding surfactants (such as LAS-type) levels in potable water. Although no specific regulations exist for biosurfactant concentrations in soil, this concentration was chosen to ensure safety and facilitate comparisons against established thresholds for chemical surfactants.

Bioremediation was executed in glass separation columns (40 × 400 mm), with each column containing 10 g of contaminated soil treated with 10 mL of biosurfactant solution. The systems were agitated at 180 rpm for 180 min to evaluate the influence of pH (ranging from 5 to 9), contact time (0–90 min), and initial contaminant concentration on glyphosate removal efficiency. Post-contact period, the exhaust valve of the separation column was opened to separate the soil from the supernatant containing the biosurfactant, which was subsequently set aside for glyphosate quantification.

Kinetic and isothermal data were determined to elucidate the adsorption mechanisms, modeled according to established equations for enzyme kinetics and bimolecular collisions [26]. Removal efficiency, expressed as percentage (%), was calculated by comparing initial (*C*_0_) and equilibrium (*Cₑ*) glyphosate concentrations in mg·L^−1^ using Equation (1):

(1) $$\mathrm{Removal}\;\left(\%\right)\;=\;\left[\frac{\left(C_{0\;}-\;C_{e}\right)}{C_{0}}\right]\;\times \;100\;$$

Additionally, the adsorption capacity (*q_e_*) was calculated using Equation (2):

(2) $$q_{e}\;=\left[\frac{\left(C_{0\;}-\;C_{e}\right)}{m}\right]\;\times \;V\;$$

where *m* is the mass of biosurfactant in kilograms, and *V* is the volume of the solution in liters.

The adsorption data were fitted to Langmuir and Temkin isotherm models, while kinetic data were analyzed through pseudo-first-order and pseudo-second-order models [26]. All experiments were performed at 26.5 °C under constant stirring at 180 rpm. The parameters of contact time and initial concentration were optimized based on preliminary analyses.

### 2.8. Tri-Acid Digestion and Xenobiotic Quantification

Given the intense adsorption of glyphosate to mineral matrices, a tri-acid digestion protocol was employed to guarantee that no “masked” xenobiotic residues remained within the remediated soil, followed by chemical derivatization and UV-Vis spectrophotometry for quantification. This rigorous oxidative process is indispensable for the exhaustive breakdown of the soil-contaminant lattice, ensuring the total liberation of bound glyphosate residues that are otherwise recalcitrant to standard extraction techniques [27]. Aliquots of the remediated soil were subjected to a concentrated mixture of nitric acid (5 mL), hydrochloric acid (2 mL), and sulfuric acid (1 mL), heated at 95 °C for three hours to ensure complete organic matter digestion and release of bound glyphosate residues. After dilution to 50 mL with ultrapure water and centrifugation, the solution was derivatized with ninhydrin to form a chromophore with an absorption peak near 570 nm. Quantification was performed via UV-Vis spectrophotometry using a calibration curve spanning from 0 to 500 µg·L^−1^, yielding an R^2^ of 0.996 for accurate interpolation of residual concentrations at µg·Kg^−1^ levels. Control experiments involved uninoculated MSM and samples treated with SDS for comparative analysis, alongside testing these samples at the same concentrations as the biosurfactant solution.

### 2.9. Phytotoxicity Bioassays

The environmental safety of the biosurfactant was monitored using lettuce *Lactuca sativa* L. (Asterales: Asteraceae) (Feltrin Sementes^®^, Farroupilha, RS, Brazil; variety Lisa, purity: 100%) as a bioindicator. A total of 25 seeds per replicate were exposed to biosurfactant concentrations (ranging from 0.00 to 4.0 mg·mL^−1^) in Petri dishes (Ø 10 cm) lined with double filter paper. Each dish was moistened with 5 mL of the biosurfactant solution and incubated at 25 °C under a 12 h photoperiod for 10 days. Control groups received an equivalent volume of sterile distilled water. Germination and root elongation (>1 cm) were assessed at 24 h intervals throughout the 10-day incubation period. The germination index (GI), relative germination (RG), and relative radicle growth (RRG) were determined using the methods outlined by Tiquia et al. [28]. The normalized residual seed germination index (NRSGI) and the normalized residual root elongation index (NRREI) were calculated as described by Bagur-González et al. [29]. Toxicity levels were categorized based on these indices: low (0 to −0.25), moderate (−0.25 to −0.5), high (−0.5 to −0.75), very high (−0.75 to −1.0), or stimulatory (hormesis effect, >0). A minimum root protrusion criterion of 1 cm was established, with radicle length measured from the hypocotyl base tip. At the conclusion of the 10 days, seedling biomass was quantified using an analytical balance with a sensitivity of 0.0001 g. The experimental design adhered to a 2 × 6 factorial arrangement (biosurfactant × concentrations), featuring 12 treatment combinations, each with five replicates of 25 seeds.

### 2.10. Statistical Analysis

Statistical analysis was conducted using Statistica© 12.5.1 software (StatSoft Inc., Tulsa, OK, USA), with a significance level set at 95%. The F-test was employed for the analysis of variance (ANOVA), assessing the variance between treatment groups against the variance within groups. Descriptive statistics were processed using OriginPro 8.5 to ensure reproducibility and reliability of the experimental results. Before analysis, data from the phytotoxicity bioassays, expressed as percentage (%), underwent arcsine transformation (x/100) 0.5 for appropriate statistical assessment.

## 3. Results

### 3.1. Valorization of Amazonian Waste and Biosurfactant Production

Proximate analysis of the babassu processing waste revealed a carbohydrate-rich matrix with a composition of 85.21% carbohydrates and a caloric value of 353.4 Kcal·100 g^−1^, establishing it as a robust, nutrient-dense substrate for the metabolic requirements of *S. ureilytica* BM01-BS. Under optimized fermentation conditions utilizing 2% (*w*/*v*) babassu waste as the sole carbon source, the strain achieved a significant biosurfactant yield of 3.7 ± 0.3 g·L^−1^. As depicted in Table 1, the biosurfactant showed impressive surface-active efficacy, reducing the surface tension to 41.80 ± 0.04 mN·m^−1^ and the oil–water interfacial tension to 6.85 ± 0.01 mN·m^−1^. Furthermore, the biosurfactant maintained a stable EI_24_ of 65.30%, which is statistically comparable to the synthetic xenobiotic surfactant SDS.

### 3.2. Genomic Architecture and Secondary Metabolite Profiling of Serratia ureilytica BM01-BS

**Figure 1:**
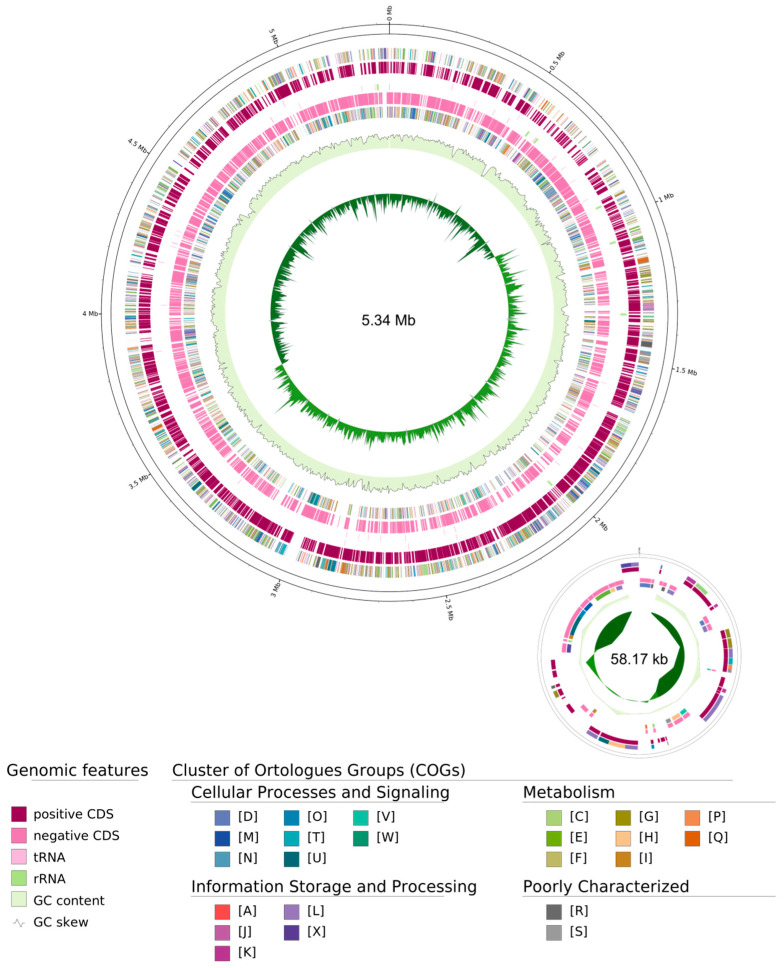
Representation of the complete genome of *Serratia ureilytica* BM01-BS. The genome consists of a circular chromosome and a circular plasmid displayed next to the chromosome (not to scale). Labelling from outside to the inside: Contigs; COGs on the forward strand; CDS, tRNAs, and rRNAs on the forward strand; CDS, tRNAs, and rRNAs on the reverse strand; COGs on the reverse strand; GC content; GC skew.

High-resolution Whole-Genome Sequencing (WGS) of *S. ureilytica* BM01-BS revealed a 5.34 Mb circular chromosome and a 58 Kb plasmid with 4996 and 63 coding sequences (CDS), respectively (Table 2; Figure 1). Taxonomic validation via the GTDB-Tk pipeline confirmed a 97% Average Nucleotide Identity (ANI) with the reference strain *S. ureilytica* CCUG:50595 (NCBI RefSeq: GCF_013375155.1). According to the data presented, the strain was classified as *S. ureilytica* BM01-BS.

As some species of *Serratia* are known for producing rhamnolipid biosurfactants, an investigation was conducted for *rhl*ABC proteins using BLASTP against characterized *Serratia rhl*ABC homologues and a custom HMMER *rhl*ABC library, based on canonical *rhl*ABC gene clusters identified by Magri and Abdel-Mawgoud [30]. Both approaches yielded no definitive homologues, with the weak hits displaying no linkage to rhamnose biosynthetic loci. Interestingly, genomic analysis via AntiSmash 8.0 identified a high-similarity Nonribosomal Peptide Synthetase (NRPS) cluster associated with the biosynthesis of a lipopeptide structurally related to Rhizomide A/B/C, featuring an N-terminal Cs (starter condensation) domain designated for fatty acid acylation (Table 3). This Whole Genome Shotgun project has been deposited in DDBJ/ENA/GenBank under the accession number JBUEBM000000000, with the version described in this paper registered as JBUEBM010000000.

### 3.3. Structural Characterization of Biosurfactant

**Figure 2:**
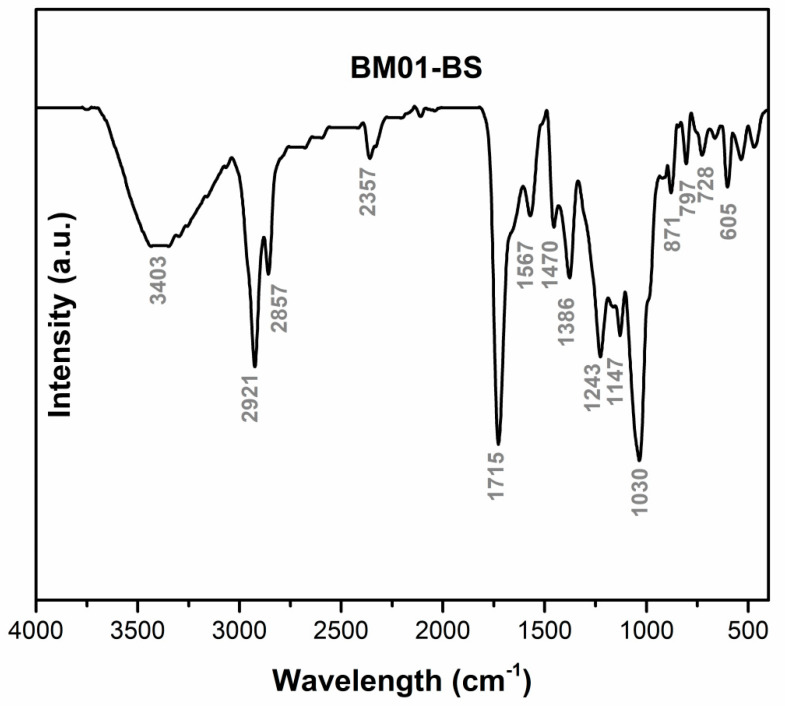
Infrared absorption spectrum (FTIR; 4000–400 cm^−1^) of the biosurfactant produced by *Serratia ureilytica* BM01-BS.

**Figure 3:**
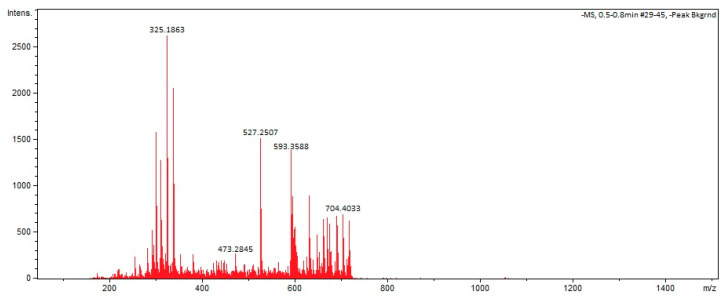
Mass spectrometry showing the *m*/*z* values of biosurfactant produced by *Serratia ureilytica* BM01-BS.

FTIR analysis of the *S. ureilytica* BM01-BS biosurfactant revealed characteristic absorption bands at 3403, 2921, 2857, 2357, 1715, 1567, 1470, 1386, 1243, 1147, 1030, 871, 797, 728, and 605 cm^−1^ (Figure 2). These findings confirmed the presence of aliphatic C–H chains (~2920 cm^−1^ and ~2850 cm^−1^), carbonyl C=O esters (~1715 cm^−1^), and glycosidic/peptide-related O–H/N–H stretching (~3400 cm^−1^). Additionally, ESI-MS fingerprinting identified a series of high-resolution ions with *m*/*z* values ranging from 220 to 721, exhibiting CH_2_-spaced homologous patterns typical of microbial glycolipid biosurfactants (Figure 3), thereby reinforcing the presence of an amphiphilic matrix.

### 3.4. Xenobiotic Bioremediation and Adsorption Dynamics

**Figure 4:**
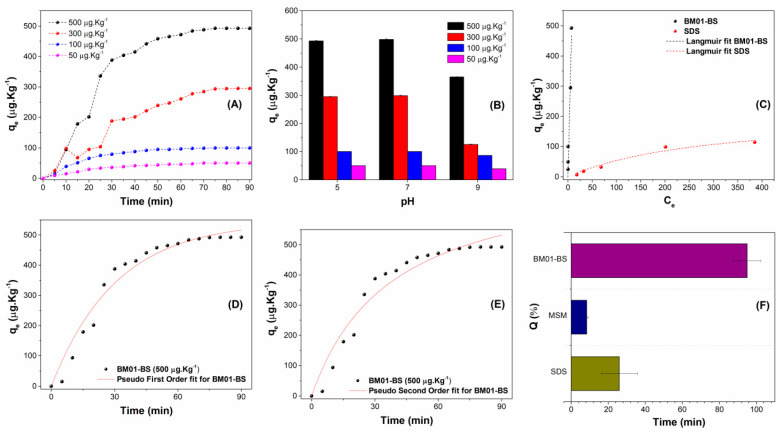
Adsorption performance of biosurfactant produced by *Serratia ureilytica* BM01-BS for glyphosate removal under different conditions: (**A**) Adsorption kinetics at different initial glyphosate concentrations (50 to 500 µg·Kg^−1^); (**B**) Effect of pH on adsorption capacity (*qₑ*) at various glyphosate concentrations; (**C**) Adsorption isotherm with Langmuir model fitting for BM01-BS and SDS (Sodium Dodecyl Sulfate); (**D**) Kinetic fit using pseudo-first-order model for BM01-BS (500 µg·Kg^−1^); (**E**) Kinetic fit using pseudo-second-order model for BM01-BS (500 µg·Kg^−1^); (**F**) Comparison of adsorption capacity (*Q*) among BM01-BS, MSM (Mineral Saline Medium), and SDS after equilibrium.

Analyzing adsorption kinetics and isotherms is crucial for understanding the mechanisms and capacity for interaction between biosurfactants (adsorbents) and contaminants. As depicted in Figure 4, increasing the initial glyphosate concentration from 50 to 500 µg·Kg^−1^ markedly enhances the adsorption capacity, with the maximum adsorption amount (*q_t_*) reaching 505.0 ± 9.2 µg·Kg^−1^ at the highest concentration tested (Figure 4A). The rapid attainment of equilibrium, observed in approximately 60 min, suggests a swift adsorption process, making it advantageous for environmental remediation applications.

The influence of pH on adsorption efficacy was assessed, revealing that optimal glyphosate removal occurs at neutral pH (7). In contrast, diminished adsorption is observed at both acidic (pH 5) and alkaline (pH 9) conditions (Figure 4B). This behavior aligns with the PZC of biosurfactant, established at 6.5. At neutral pH, the biosurfactant’s surface exhibits a near-neutral charge, minimizing electrostatic repulsions and favoring interactions such as hydrogen bonding and hydrophobic interactions. Conversely, under acidic conditions (pH 5), a positive charge emerges on the surface, leading to the repulsion of anionic glyphosate molecules, whereas under alkaline conditions (pH 9), negative predominant negative charges further diminish electrostatic interactions.

To elucidate the adsorption mechanism, sorption isotherms were modeled using the Langmuir (Equation (3)) and Temkin (Equation (4)) equations [31], with the parameters derived from these models providing insights into surface heterogeneity and binding energies, defined as follows:

(3) $$\frac{C_{e}}{q_{e}}=\frac{1}{K_{L}q_{m}}+\frac{C_{e}}{q_{m}}$$

(4) $$q_{e}=\frac{RT}{b_{T}}\ln K_{T}+\frac{RT}{b_{T}}\ln C_{e}$$

In these equations, *q_m_* (mg·Kg^−1^) represents the theoretical maximum sorption capacity of the biosurfactant, *K_L_* (L·mg^−1^) is the Langmuir isothermal constant relating the sorbent and the solute, *K_T_* (L·mol^−1^) is the Temkin isothermal constant corresponding to maximum binding energy, *b* (J·mol^−1^) is a constant related to the heat of the sorption process, *R* is the gas constant (8314 J·K^−1^ mol), and *T* (K) denotes temperature. The calculated parameters from the isotherms are summarized in Table 4, with the Langmuir isotherm plot illustrated in Figure 4C. Model fitting to the Langmuir equation yielded an R^2^ of 0.998, suggesting a monolayer adsorption process on a homogeneous surface. The theoretical maximum adsorption capacity (*q_m_*) of 845.0 ± 2.1 µg·kg^−1^ supports the high potential of biosurfactant for glyphosate remediation, reinforcing its applicability in environmental cleanup efforts. In contrast, the Temkin model provided a lower coefficient of determination (R^2^ = 0.867), suggesting that surface heterogeneity and variable binding energies are fewer dominant factors in this system.

Kinetic analysis involved two simplified models to characterize the glyphosate adsorption rate onto the biosurfactant: the pseudo-first order (Equation (5)) and pseudo-second order (Equation (6)) equations. The pseudo-first order model posits that the adsorption rate is proportional to the number of unoccupied sites, while the pseudo-second order model posits that chemisorption predominates due to valence forces. The high correlation coefficients and fit quality suggest that the pseudo-second order model more accurately describes the adsorption dynamics, indicating that chemisorption is likely the dominant mechanism. These models are defined as follows [32]:

(5) $$q_{t}=q_{e\;}\left(1-e^{-k_{1}t}\right)$$

(6) $$q_{t}=\frac{k_{2}q_{e}^{2}t}{1+k_{2}q_{e}t}$$

where *k*_1_ (1·min^−1^) and *k*_2_ (Kg·µg^−1^ min) are the rate constants associated with the kinetic models, *q_e_* (µg·Kg^−1^) denotes the amount of contaminant adsorbed per kilogram of adsorbent under experimental conditions, and *q_t_* quantifies the amount adsorbed at a specific time *t* [33]. The kinetic parameters derived from the pseudo-first order and pseudo-second order plots are detailed in Table 5.

The kinetic assessments (Figure 4D,E; Table 5) showed that the pseudo-second-order model provided a superior fit (R^2^ = 0.998) compared to the pseudo-first-order model (R^2^ = 0.899). This statistical alignment suggests that the glyphosate adsorption rate is likely influenced by rate-limiting steps involving chemical interactions at the interface, pointing toward a mechanism with chemisorption-like characteristics. Figure 4F demonstrated that the biosurfactant effectively removed >95% of glyphosate residues from contaminated soil matrices. The initial glyphosate concentration of 500 µg·kg^−1^ was reduced to a residual level of <25 µg·kg^−1^ after treatment with the BM01-BS biosurfactant. Significantly outperforming the synthetic surfactant SDS, which achieved only 20–30% removal under identical conditions.

**Figure 5:**
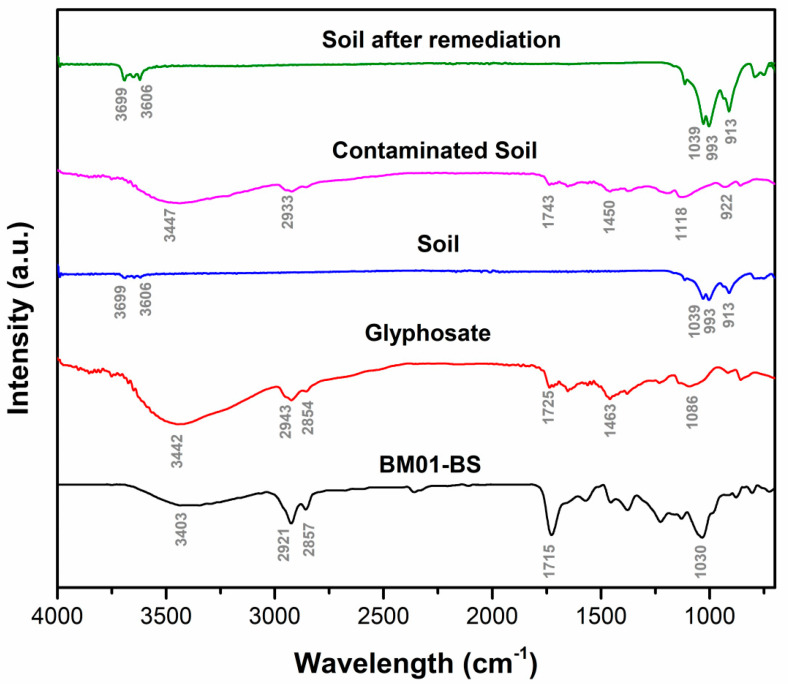
Infrared absorption spectrum (FTIR; 4000–400 cm^−1^) of biosurfactant produced by *Serratia ureilytica* BM01-BS, glyphosate, clean soil, contaminated soil (500 µg·L^−1^ glyphosate), and soil after remediation.

To complement the kinetic and isotherm analyses, FTIR served as a qualitative assessment tool to verify the efficacy of the remediation process, affirming the identification of key functional groups associated with the biosurfactant and glyphosate (Figure 5). The FTIR spectrum of the biosurfactant (black trace) exhibited a broad O–H stretching band at 3403 cm^−1^ and prominent aliphatic C–H stretching at 2921 and 2857 cm^−1^, along with a strong C=O absorption at 1715 cm^−1^ and C–O stretching near 1030 cm^−1^. The glyphosate spectrum (red trace) showed characteristic bands at 3442 cm^−1^ (O–H/N–H), 1725 cm^−1^ (C=O), and 1039–1086 cm^−1^ (P–O and C–O), indicating the presence of phosphonic and carboxylic moieties. The spectrum of contaminated soil (pink trace) displayed additional absorptions at 1743 and 1450 cm^−1^, confirming glyphosate retention, while clean soil (blue trace) revealed mineral-related bands at 3699 and 3606 cm^−1^ (O–H) and 1039 cm^−1^ (Si–O). After remediation process (green trace), the elimination of glyphosate- and biosurfactant-related peaks (1725, 1463, and 2921 cm^−1^) alongside the recovery of mineral bands (1039 and 913 cm^−1^) indicated complete removal of both the contaminant and the biosurfactant, restoring the chemical integrity of the soil matrix.

### 3.5. Ecotoxicological Monitoring and Environmental Safety

Phytotoxicity evaluations utilizing *Lactuca sativa* demonstrated that the biosurfactant exhibited a favorable environmental safety profile across all tested concentrations (0.01 to 4.0 mg·mL^−1^) (Table 6). The Germination Index (GI) exceeded the 80% non-phytotoxic threshold, while the Normalized Residual Seed Germination Index (NRSGI) and Normalized Residual Root Elongation Index (NRREI) fell within the “low toxicity” range (0 to −0.25). These findings demonstrate that the bioremediation process effectively restores soil health and ecological resilience without introducing secondary xenobiotic stressors.

## 4. Discussion

The potential for utilizing babassu waste as a primary substrate in fermentation processes is indicated by its nutritional composition, which includes a carbohydrate content of 85.21% [12]. This remains below the acceptable thresholds set by Brazilian standards for plant-derived products [34]. Beyond waste valorization, these findings contribute to an innovative platform that enables the production of secondary metabolites while circumventing the economic drawbacks associated with petroleum-derived substrates including glycerol [35]. The biosurfactant yield from *S. ureilytica* BM01-BS, measured at 3.7 ± 0.3 g·L^−1^, surpasses that of conventional strains such as *P. aeruginosa* [36] under similar conditions. Despite thorough research, we were unable to identify any studies assessing the biosurfactant production yields of *Serratia* strains. This lack of data precludes meaningful comparisons of our findings with existing literature under similar conditions. The notable metabolic efficiency of this Amazonian isolate, adapted to thrive in high-stress, mineral-rich environments [37], favors the production of biosurfactants with high emulsification stability (EI_24_ > 65%) and substantial reductions in surface and interfacial tensions, corroborating the previous findings [38]. The energy value of the biomass waste (353.4 Kcal·100 g^−1^) further validates its nutritional and biotechnological potential [39].

Although the biosurfactant yield from *S. ureilytica* BM01-BS did not align with preliminary expectations (>5 g·L^−1^), optimization of the fermentation process is warranted. Critical parameters such as oxygenation levels in the culture medium, microbial growth dynamics, and bioprocess strategies (batch versus fed-batch) require thorough examination to fully unlock the biosurfactant production capacity of this strain. However, the use of babassu waste as a renewable and cost-effective raw material positions this approach favorably within the context of enhancing market competitiveness and diminishing dependence on glycerol. This strategy aligns with global initiatives aimed at promoting circular economies and valorizing agro-industrial waste streams, particularly in the Amazon region, fostering regional sustainability and socio-economic development.

Although the chemical identity of the *S. ureilytica* BM01-BS biosurfactant has not been unambiguously established, preliminary spectroscopic data, along with genome analysis, provides evidence of its probable composition. FTIR (Figure 2) and ESI-MS (Figure 3) fingerprints initially suggested a glycolipid-type structure, characterized by aliphatic chains and carbonyl groups [40,41]; however, high-resolution WGS followed by AntiSmash 8.0 analysis (Table 2; Figure 1) provided a critical counterpoint. Importantly, FTIR lacks the chemical resolution necessary to distinguish between glycolipid and lipopeptide scaffolds, particularly in the absence of clearly resolved amide I bands or sugar-specific diagnostic patterns [42]. Similarly, while our ESI-MS data reveal ions within mass ranges commonly reported for mono- and di-rhamnolipids, these findings do not constitute definitive evidence for the presence of molecules of this type. The absence of canonical rhamnolipid biosynthetic genes (*rhlABC*) in the *S. ureilytica* BM01-BS genome, coupled with the identification of a BGC with high-similarity to nonribosomal peptide synthetase (NRPS) clusters related to Rhizomide A/B/C, strongly suggests that the biosurfactant produced by *S. ureilytica* BM01-BS is of lipopeptide origin. Lipopeptide biosurfactants consist of a hydrophobic fatty acid moiety linked to a hydrophilic peptide chain [43]. The predicted product of the NRPS cluster is a hydrophilic Thr-Ser-Ser-Ile-Val pentapeptide chain. Furthermore, the identification of an N-terminal Cs domain in the NRPS is indicative of lipopeptides, as these domains facilitate the transfer of a fatty acid to the initial amino acid in the peptide chain [44].

Based on our findings, we hypothesize that a nonribosomal lipopentapeptide contributes to the observed surface activity and the high removal efficiency of glyphosate by the *S. ureilytica* BM01-BS biosurfactant. Although this hypothesis warrants further investigation, it is supported by reports of other strains within this species which generate biosurfactants and which possess homologous NRPS clusters. Although the current data strongly supports the “Rhizomide Hypothesis,” definitive structural elucidation of individual congeners using tandem mass spectrometry (MS/MS) and nuclear magnetic resonance (NMR) is an immediate priority for future investigations. Additionally, a definitive experimental link between this biosynthetic gene cluster and the produced metabolite has yet to be established. Future investigations employing targeted gene knockouts or transcriptomic analysis under fermentative stress are essential to definitively validate the functional role of this NRPS cluster in glyphosate remediation. This conservative interpretation maintains the study’s focus on the metabolic potential of Amazonian isolates while identifying the necessary milestones for future structural and functional genomics.

From a xenobiotic perspective, distinguishing between lipopeptides and glycolipids is crucial, as lipopeptides typically exhibit greater structural complexity and functional diversity, potentially providing advanced mechanisms for the chelation or entrapment of persistent herbicides like glyphosate [45]. The biosurfactant produced by *S. ureilytica* BM01-BS demonstrates a remarkable glyphosate removal rate exceeding 95% within approximately 60 min, marking significant progress in addressing this persistent anthropogenic xenobiotic (Figure 4). The superior statistical fit of the experimental data to the pseudo-second-order kinetic model (Table 5) suggests that the adsorption rate may be governed by a rate-limiting step involving chemical interactions, rather than simple physical diffusion. This behavior points toward a mechanism where the interaction between glyphosate and the soil-biosurfactant interface likely involves valence forces, such as hydrogen bonding or ligand exchange, aligning with previous studies on the remediation of organic xenobiotics. However, it is important to note that these kinetic models provide an empirical description of the adsorption rate, and the high correlation with the pseudo-second-order model serves as a mechanistic indicator of chemisorption-like behavior in the system [46].

The adsorption efficiency of the biosurfactant varies according to factors such as pH, temperature, initial glyphosate concentration, and surface modifications of the adsorbent [47]. Notably, the solution’s pH influences the ionization states of both glyphosate and the biosurfactant, affecting the overall adsorption capacity [48]. The thermodynamic alignment between the PZC (6.5) and the optimal remediation pH (7.0) elucidates this mechanism; at near-neutral pH, the biosurfactant maintains charge neutrality, minimizing electrostatic repulsion with the glyphosate’s anionic phosphonate groups. This “electrostatic window” allows for maximum interfacial affinity, explaining why the biosurfactant dramatically outperformed the synthetic surfactant SDS, which achieved only 20–30% glyphosate removal due to its linear structure and insufficient complex functional groups to disrupt glyphosate-soil mineral interactions.

The qualitative validation of the remediation process through FTIR spectral analysis (Figure 5) provides a “chemical signature” indicative of success. The complete disappearance of P–O and C=O stretching bands, along with the restoration of native mineral Si–O bands in the remediated soil spectrum, demonstrate a full recovery of the soil’s chemical integrity. These spectral shifts underscore the viability of FTIR as an efficient qualitative technique for monitoring the progress, supporting non-destructive and rapid assessments, as corroborated by Chakraborty et al. [49] and Liu et al. [50] observations. Additionally, the non-toxic effects observed on plant health further advocate for the environmental safety of employing biosurfactant in natural settings, enhancing its appeal for field applications.

The phytotoxicity trials using *Lactuca sativa* (Table 6) confirmed the biosurfactant’s biological safety, as Germination Index (GI) values ranged from 86% to 100%, consistently exceeding the non-phytotoxic threshold [51]. The low toxicity scores (NRSGI and NRREI) further validate the isolated biosurfactant as a safe, bio-rational adjuvant for bioremediation of xenobiotics. By correlating genomic insights with applied environmental chemistry, this research advances the goals of a circular bioeconomy by offering a solution for the persistent issue of herbicide contamination, corroborating previous studies regarding the versatility of biosurfactants in environmental cleanup operations [52,53]. Moreover, the unique combination of biodegradability, low toxicity, and surface-active capabilities makes biosurfactants increasingly attractive for commercial applications [41].

The global market for biosurfactants is projected to reach approximately US$14.7 billion in 2035 [54], with biosurfactant-based processes potentially reducing production costs by up to 50% through the use of alternative, cost-effective raw materials, such as agro-industrial processing waste [55]. This approach promotes waste stream valorization [35,56] and aligns with health policies advocating the use of sustainable, plant-based inputs [57,58]. Given the growing demand for environmentally friendly biotechnological solutions, the findings of this study position the biosurfactants produced by the BM01-BS strain as promising candidates for large-scale application in the bioremediation of glyphosate-contaminated soils.

## 5. Conclusions

This study validates an integrated bioremediation framework using specialized biosurfactants derived from *Serratia ureilytica* BM01-BS, produced through the sustainable valorization of Amazonian babassu agro-industrial waste. By using carbohydrate-rich biomass (85.21%) as a sole nutrient source, we have established a cost-effective bioprocess that achieves a significant biosurfactant yield (3.7 g·L^−1^) capable of removing >95% of glyphosate residues from contaminated soil matrices within 60 min, rendering a bioremediation process both technically superior and economically viable to commercial toxic surfactants. From a mechanistic perspective, the adsorption dynamics were well-described by a pseudo-second-order kinetic model, suggesting that the removal of this persistent xenobiotic is likely influenced by chemical interactions at the interface, characteristic of a chemisorption-like process. This high-affinity interaction is optimized at a neutral pH (7.0), aligning with the biosurfactant’s Point of Zero Charge (6.5) to minimize electrostatic repulsion and facilitate ligand exchange with the xenobiotic. Furthermore, genomic analysis suggests that the high affinity for glyphosate is linked to specialized lipopentapeptide scaffolds.

Qualitative validation via FTIR and ecotoxicological monitoring using *Lactuca sativa* confirmed the total efficacy and environmental safety of this process. The complete disappearance of glyphosate-associated phosphonic and carboxylic bands, coupled with the recovery of native soil mineral bands, proves that the bioremediation process effectively restores the chemical and ecological integrity of the impacted landscape. Ultimately, this research establishes a scalable, biodiversity-based framework that transcends traditional high-cost bioprocessing, ensuring that the mitigation of persistent xenobiotic legacies aligns with the pragmatic requirements of a sustainable global circular bioeconomy. To unlock the full potential of biosurfactant-based bioremediation, future research should focus on optimizing production processes, including scaling strategies and comprehensive environmental impact assessments to facilitate the transition from controlled laboratory conditions to practical field applications to mitigate widespread environmental pollution.

## Figures and Tables

**Table 1:** Physicochemical properties of biosurfactant produced by *Serratia ureilytica* BM01-BS.

Properties	BM01-BS	Controls
Emulsification index (%)	65.30 ± 1.44	66.00 ± 1.30 ^a^
Surface tension (mN·m^−1^)	41.80 ± 0.04	72.01 ± 0.08 ^b^
Interfacial tension (mN·m^−1^)	6.85 ± 0.01	17.00 ± 0.04 ^c^

^a^: 1% sodium dodecyl sulfate (SDS) was used as a control for emulsification tests; ^b^: Distilled water was used as a control for surface tension tests; ^c^: Semi-synthetic lubricating oil was used as a control for interfacial tension tests. Values were determined at a biosurfactant concentration of 10 mg·mL^−1^, and results were expressed as mean ± standard deviation of three replicates (n = 3).

**Table 2:** Features of *Serratia ureilytica* str. BM01-BS genome.

Feature	Metric *
Sequence length (bp)	5,337,710 (58,171)
Number of contigs	2
GC content %	59.1
Number of CDS	4996 (63)
rRNA operons	22
tRNA operons	90
tmRNAs	1
ncRNAs	54
CRISPR arrays	0
BUSCO ^†^	C: 96.8% [S: 96.0%, D: 0.8%], F: 0.0%, M: 3.2%, n: 124

* Number in brackets refers to the plasmid. ^†^ Benchmarking Universal Single-Copy Orthologue: complete (C), complete and single-copy (S), complete and duplicated (D), Fragmented (F), Missing (M), total searched (n).

**Table 3:** Secondary metabolite prediction for *Serratia ureilytica* BM01-BS.

Type	From	To	Similarity Confidence	Most Similar Known Cluster
Betalactone	106,165	131,833		
NRP-metallophore, NRPS	504,224	568,291	Low	trichrysobactin/cyclic trichrysobactin/chrysobactin/dichrysobactin
hserlactone	836,201	856,875		
NRPS, T1PKS	1,132,634	1,187,256		Rhizomide A/B/C
NRPS	1,408,816	1,466,668	High	kolossin
arylpolyene, NRPS-like	2,787,867	2,837,320	High	andrimid
NRPS	2,854,154	2,930,892	Low	5-dimethylallylindole-3-acetonitrile
NRPS	3,589,642	3,638,152	Low	viobactin
RRE-containing	3,656,761	3,677,039		
opine-like-metallophore	3,965,068	3,987,165	High	yersinopine
azole-containing-RiPP	4,381,157	4,407,599		
terpene-precursor	5,091,833	5,112,753		

**Table 4:** Isotherms parameters calculated for glyphosate adsorption by biosurfactant produced by *Serratia ureilytica* BM01-BS at 25 °C and 500 µg·L^−1^.

Isotherm Model	Parameter	Glyphosate/BM01-BS
Langmuir	*q_m_* (mg·g^−1^)	845.0 ± 2.1
	*K_L_* (L·mg^−1^)	0.775
	R^2^	0.998
Temkin	*K_T_* (L·mg^−1^)	655.85 ± 5.6
	* b_T_*	0.701
	R^2^	0.867

*q_m_* (theoretical maximum sorption capacity of the biosurfactant), *K_L_* (Langmuir isothermal constant relating the sorbent and the solute), *K_T_* (Temkin isothermal constant corresponding to maximum binding energy), *b* (constant related to the heat of the sorption process), *R* (gas constant: 8314 J·K^−1^ mol), and *T* (temperature in K).

**Table 5:** Kinetic parameters calculated for glyphosate adsorption by biosurfactant produced by *Serratia ureilytica* BM01-BS at 25 °C.

*C*_0_	*q_e_*_(*exp*)_	*q_e_*_(*calc*)_	*k*_1_ (×10^−3^)	R^2^
Pseudo-first order				
50	68.1	78.1	0.4	0.856
100	145.2	154.2	0.4	0.854
300	398.5	419.3	0.6	0.897
500	756.0	815.0	0.9	0.899
Pseudo-second order				
50	79.8	80.5	0.1	0.927
100	152.3	160.4	0.8	0.937
300	391.5	425.5	1.0	0.950
500	816.9	845.0	1.5	0.998

*C*_0_ (initial equilibrium glyphosate concentrations), *q_e_*_(*exp*)_ (amount of contaminant adsorbed per gram of adsorbent), *q_e_*_(*calc*)_ (theoretical values derived from the kinetic models), and *k*_1_ (rate constant associated with the kinetic models). *C*_0_, *q_e_*_(*exp*)_, and *q_e_*_(*calc*)_ are expressed in µg·kg^−1^, and *k*_1_ is expressed in 1·min^−1^.

**Table 6:** Effect of exposure to biosurfactant on *Lactuca sativa* L.

Biosurfactant (mg·mL^−1^)	Parameters							
	Germ *	Len	NRSGI *	NRREI	RRG *	RG *	GI	BP *
0.00	100	1.95 ^a^	0.00	−0.03 ^a^	100	100	100 ^a^	0.175
0.01	100	1.90 ^a^	−0.08	−0.05 ^a^	97	96	94 ^a,b^	0.156
0.1	100	1.74 ^b^	0.00	−0.15 ^b^	87	100	87 ^b^	0.187
1.0	100	1.74 ^b^	−0.01	−0.13 ^b^	89	98	88 ^b^	0.302
2.0	100	1.70 ^b^	−0.01	−0.15 ^b^	87	100	86 ^b^	0.256
4.0	100	1.70 ^b^	0.00	−0.15 ^b^	87	100	87 ^b^	0.257

Results are expressed as mean ± standard deviation (n = 5). According to the Tukey test, means with the same letters, when arranged vertically, are considered to belong to the same group with a 5% probability. Percentage data have been transformed using the equation (x + 1)0.5. The 0.00 mg·mL^−1^ concentration denotes the control group, where *Lactuca sativa* seeds were germinated in distilled water. Germ (germinability), RRG (relative radicle growth), RG (relative germination), and GI (germination index) are expressed in %; Len: Length, expressed in cm; NRSGI: normalized residual seed germination index; NRREI: normalized residual root elongation index; BP: biomass plant, expressed in g; *: no significant differences.

## Data Availability

*S. ureilytica* BM01-BS genome data are openly available in National Center for Biotechnology Information at https://www.ncbi.nlm.nih.gov/bioproject/PRJNA1416668 (accessed on 10 March 2026).

## Abbreviations

The following abbreviations are used in this manuscript:

CFB	Cell-Free Broth
ESI-MS	Electrospray Ionization Mass Spectrometry
EI_24_	Emulsification Index
FTIR	Fourier-Transform Infrared Spectroscopy
GI	Germination Index
MSM	Mineral Saline Medium
NRPS	Nonribosomal Peptide Synthetase
NRREI	Normalized Residual Root Elongation Index
NRSGI	Normalized Residual Seed Germination Index
PZC	Point of Zero Charge
RG	Relative Germination
RRG	Relative Radicle Growth
WGS	Whole-Genome Sequencing

## References

[1] Mazuryk J., Klepacka K., Kutner W., Sharma P.S. (2024). Glyphosate: Hepatotoxicity, Nephrotoxicity, Hemotoxicity, Carcinogenicity, and Clinical Cases of Endocrine, Reproductive, Cardiovascular, and Pulmonary System Intoxication. ACS Pharmacol. Transl. Sci..

[2] Aoun P.G., Khairallah W., Rejeb A., Haddarah A. (2025). Glyphosate Use in Crop Systems: Risks to Health and Sustainable Alternatives. Toxics.

[3] Gentile M., Luengo C., Xiong J., Wang M., Tan W., Avena M. (2025). Glufosinate, Glyphosate and Phosphate. Understanding Their Adsorption Trends on a Mineral Surface. Sci. Total Environ..

[4] Adeola A.O., Paramo L., Duarte M.P., Fuoco G., Naccache R. (2025). Unraveling Glyphosate Sequestration: The Role of Natural Organic Matter Fractions in Soil-Water Contamination and Retention. J. Environ. Manag..

[5] Alves J.A., Cruz da Silva E., Sá G.C.S., Moura Feio A., Oliveira Ramos E., Soares Gomes G., De Siqueira Pimentel L.M., Cerqueira dos Santos S. (2025). Biosurfactant production by *Pseudomonas aeruginosa* using cocoa (*Theobroma cacao* L.) residue and its potential in oil dispersion. Biodiversidade Bras..

[6] Cano-Gómez C.I., Wong-Arguelles C., Hinojosa-López J.I., Muñiz-Márquez D.B., Wong-Paz J.E. (2025). Novel Insights into Agro-Industrial Waste: Exploring Techno-Economic Viability as an Alternative Source of Water Recovery. Waste.

[7] Wang M., Ding M., Yuan Y. (2023). Bioengineering for the Microbial Degradation of Petroleum Hydrocarbon Contaminants. Bioengineering.

[8] Zhang M., Chen Q., Gong Z. (2024). Microbial Remediation of Petroleum-Contaminated Soil Focused on the Mechanism and Microbial Response: A Review. Environ. Sci. Pollut. Res. Int..

[9] Renganathan P., Gaysina L.A. (2025). Next-Generation Wastewater Treatment: Omics and AI-Driven Microbial Strategies for Xenobiotic Bioremediation and Circular Resource Recovery. Processes.

[10] Zhang Y., Sasaki J., Li A., Chen J. (2025). Application and Challenges of Machine Learning in Microbial Remediation: A Review of Current Status and Future Directions. Crit. Rev. Environ. Sci. Technol..

[11] Selim S., Harun-Ur-Rashid M. (2025). Harnessing Microbial Diversity for Effective Remediation of Heavy Metal-Contaminated Soils. Appl. Soil Ecol..

[12] Feio A.M., Sá G.C.S., Orsato A., Leite K., Pimentel L.M.S., Alves J.d.A., Gomes G.S., Ramos E.O., Quintella C.M., Fragoso S.P. (2025). Valorization of Amazonian Fruit Biomass for Biosurfactant Production and Nutritional Applications. Biomass.

[13] De Coster W., D’Hert S., Schultz D.T., Cruts M., Van Broeckhoven C. (2018). NanoPack: Visualizing and Processing Long-Read Sequencing Data. Bioinformatics.

[14] Kolmogorov M., Yuan J., Lin Y., Pevzner P.A. (2019). Assembly of Long, Error-Prone Reads Using Repeat Graphs. Nat. Biotechnol..

[15] Vaser R., Šikić M. (2021). Time- and Memory-Efficient Genome Assembly with Raven. Nat. Comput. Sci..

[16] Chen Y., Nie F., Xie S.-Q., Zheng Y.-F., Dai Q., Bray T., Wang Y.-X., Xing J.-F., Huang Z.-J., Wang D.-P. (2021). Efficient Assembly of Nanopore Reads via Highly Accurate and Intact Error Correction. Nat. Commun..

[17] Chaumeil P.-A., Mussig A.J., Hugenholtz P., Parks D.H. (2022). GTDB-Tk v2: Memory Friendly Classification with the Genome Taxonomy Database. Bioinformatics.

[18] Tatusova T., DiCuccio M., Badretdin A., Chetvernin V., Nawrocki E.P., Zaslavsky L., Lomsadze A., Pruitt K.D., Borodovsky M., Ostell J. (2016). NCBI Prokaryotic Genome Annotation Pipeline. Nucleic Acids Res..

[19] Simão F.A., Waterhouse R.M., Ioannidis P., Kriventseva E.V., Zdobnov E.M. (2015). BUSCO: Assessing Genome Assembly and Annotation Completeness with Single-Copy Orthologs. Bioinformatics.

[20] Blin K., Shaw S., Vader L., Szenei J., Reitz Z.L., Augustijn H.E., Cediel-Becerra J.D.D., de Crécy-Lagard V., Koetsier R.A., Williams S.E. (2025). AntiSMASH 8.0: Extended Gene Cluster Detection Capabilities and Analyses of Chemistry, Enzymology, and Regulation. Nucleic Acids Res..

[21] Cumsille A., Durán R.E., Rodríguez-Delherbe A., Saona-Urmeneta V., Cámara B., Seeger M., Araya M., Jara N., Buil-Aranda C. (2023). GenoVi, an Open-Source Automated Circular Genome Visualizer for Bacteria and Archaea. PLoS Comput. Biol..

[22] Bodour A.A., Miller-Maier R.M. (1998). Application of a Modified Drop-Collapse Technique for Surfactant Quantitation and Screening of Biosurfactant-Producing Microorganisms. J. Microbiol. Methods.

[23] Das M., Das S.K., Mukherjee R.K. (1998). Surface Active Properties of the Culture Filtrates of a *Micrococcus* Species Grown on n-Alkanes and Sugars. Bioresour. Technol..

[24] Sá G.C.S., Bezerra P.V.V., Ramos E.O., Orsato A., Leite K., Feio A.M., Pimentel L.M.S., Alves J.A., Gomes G.S., Rodrigues P.D. (2025). *Pseudomonas aeruginosa* Rhamnolipids Produced by Andiroba (*Carapa guianensis* Aubl.) (Sapindales: Meliaceae) Biomass Waste from Amazon: A Potential Weapon Against *Aedes aegypti* L. (Diptera: Culicidae). Molecules.

[25] Bakatula E.N., Richard D., Neculita C.M., Zagury G.J. (2018). Determination of Point of Zero Charge of Natural Organic Materials. Environ. Sci. Pollut. Res. Int..

[26] Silva E.C., Soares V.R., Fajardo A.R. (2023). Removal of Pharmaceuticals from Aqueous Medium by Alginate/Polypyrrole/ZnFe_2_O_4_ Beads via Magnetic Field Enhanced Adsorption. Chemosphere.

[27] Elbagory M., Zayed A., El-Khateeb N., El-Nahrawy S., Omara A.E.D., Mohamed I., Elbyaly M.Y.H., El-Sharkawy M., Singh J., Dzaja A. (2025). Risk Assessment of Potentially Toxic Heavy Metals in Wheat (*Triticum aestivum* L.) Grown in Soils Irrigated with Paper Mill Effluent. Toxics.

[28] Tiquia S.M., Tam N.F.Y., Hodgkiss I.J. (1996). Effects of Composting on Phytotoxicity of Spent Pig-Manure Sawdust Litter. Environ. Pollut..

[29] Bagur-González M.G., Estepa-Molina C., Martín-Peinado F., Morales-Ruano S. (2011). Toxicity Assessment Using *Lactuca sativa* L. Bioassay of the Metal(Loid)s As, Cu, Mn, Pb and Zn in Soluble-in-Water Saturated Soil Extracts from an Abandoned Mining Site. J. Soils Sediments.

[30] Magri M., Abdel-Mawgoud A.M. (2022). Identification of Putative Producers of Rhamnolipids/Glycolipids and Their Transporters Using Genome Mining. Curr. Res. Biotechnol..

[31] Ngo T.S., Tracey C.T., Navrotskaya A.G., Bukhtiyarov A.V., Krivoshapkin P.V., Krivoshapkina E.F. (2023). Reusable Carbon Dot/Chitin Nanocrystal Hybrid Sorbent for the Selective Detection and Removal of Cr(VI) and Co(II) Ions from Wastewater. Carbohydr. Polym..

[32] Badsha M.A.H., Khan M., Wu B., Kumar A., Lo I.M.C. (2021). Role of Surface Functional Groups of Hydrogels in Metal Adsorption: From Performance to Mechanism. J. Hazard. Mater..

[33] Kavand M., Asasian N., Soleimani M., Kaghazchi T., Bardestani R. (2017). Film-Pore-[Concentration-Dependent] Surface Diffusion Model for Heavy Metal Ions Adsorption: Single and Multi-Component Systems. Process Saf. Environ. Prot..

[34] (1978). Special Technical Norms Relating to Food and Beverages for Use throughout Brazilian Territory.

[35] Zhu H., Zhang F., Hu J., Zhang Z., Zhang R. (2025). Microbial Valorization of Food Waste into Biosurfactants: Innovations and Application in Food Industry. Crit. Rev. Food Sci. Nutr..

[36] Zhao F., Yuan M., Lei L., Li C., Xu X. (2021). Enhanced Production of Mono-Rhamnolipid in *Pseudomonas aeruginosa* and Application Potential in Agriculture and Petroleum Industry. Bioresour. Technol..

[37] Sousa N.S.O., Souza E.S., Canto E.S.M., Silva J.P.A., Carneiro L.M., Franco-de-Sá J.F.O., Souza J.V.B. (2023). Amazonian Fermentations: An Analysis of Industrial and Social Technology as Tools for the Development of Bioeconomy in the Region. Braz. J. Biol..

[38] Pele M.A., Ribeaux D.R., Vieira E.R., Souza A.F., Luna M.A.C., Rodríguez D.M., Andrade R.F.S., Alviano D.S., Alviano C.S., Barreto-Bergter E. (2019). Conversion of Renewable Substrates for Biosurfactant Production by *Rhizopus arrhizus* UCP 1607 and Enhancing the Removal of Diesel Oil from Marine Soil. Electron. J. Biotechnol..

[39] Sá G.C.S., Silva M.A.T., Silva D.F., Santi-Gadelha T., Fragoso S.P., Madruga M.S., Pacheco M.T.B., Lima E.O., Uchôa A.F., Gadelha C.A.A. (2021). Nutritional Composition and Biological Activities (Antioxidant and Antifungal) of *Sesbania virgata* (Cav.) Pers. Seeds. Rev. Bras. Tecnol. Agroind..

[40] Deepika K.V., Kalam S., Ramu Sridhar P., Podile A.R., Bramhachari P.V. (2016). Optimization of Rhamnolipid Biosurfactant Production by Mangrove Sediment Bacterium *Pseudomonas aeruginosa* KVD-HR42 Using Response Surface Methodology. Biocatal. Agric. Biotechnol..

[41] Santos S.C., Torquato C.A., Santos D.d.A., Orsato A., Leite K., Serpeloni J.M., Losi-Guembarovski R., Pereira E.R., Dyna A.L., Barboza M.G.L. (2024). Production and Characterization of Rhamnolipids by *Pseudomonas aeruginosa* Isolated in the Amazon Region, and Potential Antiviral, Antitumor, and Antimicrobial Activity. Sci. Rep..

[42] Darwiche N., Dufresne C., Chartier A., Claude B., Colas C., Fougère L., Sebban M., Lucchesi M.E., Le Floch S., Nehmé R. (2025). Glycolipid and Lipopeptide Biosurfactants: Structural Classes and Characterization—Rhamnolipids as a Model. Crit. Rev. Anal. Chem..

[43] Dini S., Bekhit A.E.D.A., Roohinejad S., Vale J.M., Agyei D. (2024). The Physicochemical and Functional Properties of Biosurfactants: A Review. Molecules.

[44] Antonioli Júnior R., Poloni J.d.F., Pinto É.S.M., Dorn M. (2022). Interdisciplinary Overview of Lipopeptide and Protein-Containing Biosurfactants. Genes.

[45] Mnif I., Ghribi D. (2015). Lipopeptides Biosurfactants: Mean Classes and New Insights for Industrial, Biomedical, and Environmental Applications. Biopolymers.

[46] Zhang M., Chen W., Chuan X., Guo X., Shen X., Zhang H., Wu F., Hu J., Wu Z., Wang X. (2024). Remediation of Heavily PAHs-Contaminated Soil with High Mineral Content from a Coking Plant Using Surfactant-Enhanced Soil Washing. Sci. Total Environ..

[47] Souza K.S., da Silva M.R.F., Candido M.A., Lins H.T.S., de Lima Torres G., da Silva Felix K.C., Silva K.C.C., Filho R.M.N., Bhadouria R., Tripathi S. (2025). Biodegradation Potential of Glyphosate by Bacteria: A Systematic Review on Metabolic Mechanisms and Application Strategies. Agronomy.

[48] Briceño S., Arevalo-Fester J.E. (2025). Sustainable Nanomaterials for Glyphosate Remediation. ACS Appl. Eng. Mater..

[49] Chakraborty J., Das S. (2017). Application of Spectroscopic Techniques for Monitoring Microbial Diversity and Bioremediation. Appl. Spectrosc. Rev..

[50] Liu J., Li W., Ren X., Qi Z., Ma J., Huang S., Chai L., Jiao Y., Xu J., Liu X. (2025). Study on the Bioremediation of Methylene Blue by *Haematococcus pluvialis* through Synchrotron-FTIR Imaging and Spectroscopy. Spectrochim. Acta A Mol. Biomol. Spectrosc..

[51] EPA Ecological Effects Test Guidelines (1996). OPPTS 850.4200: Seed Germination/Root Elongation Toxicity Test.

[52] Abis L.A., Hassan O.M., Hassan K.T. (2025). Biosurfactants in the Remediation of Petroleum-Contaminated Soils: Mechanisms and Applications. Ecol. Eng. Environ. Technol..

[53] Pandit N.K., Meena S.S. (2025). Exploring Sustainable Biosurfactant Production Through Waste Valorization: Emerging Research Trends and Industrial Applications. Waste Biomass Valorization.

[54] GMI Global Market Insights—Biosurfactants Market Size & Share 2026–2035. https://www.gminsights.com/industry-analysis/biosurfactants-market-report.

[55] Silva R., Almeida D., Rufino R., Luna J., Santos V., Sarubbo L. (2014). Applications of Biosurfactants in the Petroleum Industry and the Remediation of Oil Spills. Int. J. Mol. Sci..

[56] Qamar S.A., Pacifico S. (2023). Cleaner Production of Biosurfactants via Bio-Waste Valorization: A Comprehensive Review of Characteristics, Challenges, and Opportunities in Bio-Sector Applications. J. Environ. Chem. Eng..

[57] Sá G.C.S., da Silva L.B., Bezerra P.V.V., da Silva M.A.F., Inacio C.L.S., Paiva W.d.S., e Silva V.P.M., Cordeiro L.V., Oliveira J.W.d.F., Silva M.S. (2023). *Tephrosia Toxicaria* (Sw.) Pers. Extracts: Screening by Examining Aedicidal Action under Laboratory and Field Conditions along with Its Antioxidant, Antileishmanial, and Antimicrobial Activities. PLoS ONE.

[58] Brazilian Policy on Medicinal Plants and Herbal Medicines [Portuguese]. https://www.planalto.gov.br/ccivil_03/_Ato2004-2006/2006/Decreto/D5813.htm.

